# Use of Pseudocereals Preferment Made with Aromatic Yeast Strains for Enhancing Wheat Bread Quality

**DOI:** 10.3390/foods8100443

**Published:** 2019-09-26

**Authors:** Adriana Păucean, Simona Maria Man, Maria Simona Chiş, Vlad Mureşan, Carmen Rodica Pop, Sonia Ancuţa Socaci, Crina Carmen Mureşan, Sevastiţa Muste

**Affiliations:** 1 Department of Food Engineering, Faculty of Food Science and Technology, University of Agricultural Sciences and Veterinary Medicine of Cluj-Napoca, 3-5, Manastur Street, 400372 Cluj-Napoca, Romania; adriana.paucean@usamvcluj.ro (A.P.); simona.chis@usamvcluj.ro (M.S.C.); vlad.muresan@usamvcluj.ro (V.M.);; 2 Department of Food Science, Faculty of Food Science and Technology, University of Agricultural Sciences and Veterinary Medicine of Cluj-Napoca, 3-5, Manastur Street, 400372 Cluj-Napoca, Romania

**Keywords:** quinoa, aromatic yeast, preferment, wheat bread

## Abstract

Usually, aromatic yeasts are designed to ferment wheat substrates for baking purposes but identification of new substrates for these strains and consequently new formulations for dough could lead to diversified bakery products with improved nutritional qualities and specific sensorial properties. The purpose of our study was to optimize the fermentation of quinoa and amaranth flours with non-conventional yeast strains in order to obtain a preferment with high potential in enhancing nutritional, textural and sensorial features of white wheat bread. Two biotypes of *Saccharomyces cerevisiae* yeast—a wine yeast strain and a beer yeast strain—commercialized for their aromatic properties were used. Both aromatic yeast strains revealed good performance on fermenting pseudocereal substrates. Utilization of the obtained preferment in white wheat breadmaking led to bread with higher protein, fibres, mineral, total polyphenols content, with specific texture and aroma profile and high consumers’ acceptability.

## 1. Introduction

Nowadays, the development of new bakery products based on pseudocereals is considered to be a necessity rather than a choice. The attention toward these ancient grains has been renewed by the increasing demand for natural and health-beneficial food. From a nutritional point of view, reintroducing pseudocereals in the daily diet as a fortifying agent with functional added-value features, might offer to consumers a richer variety of beneficial compounds without altering the technological quality [1].

Among ancient grains, quinoa (*Chenopodium quinoa* Wild, family *Amaranthaceae*) is a rich source of minerals, vitamins, fibers, fatty acids and has well-balanced protein and amino acid content that could develop dietary protein balance when utilized by it or mixed with cereal grains [2]. It is high in magnesium and iron contents as well as in vitamins such as vitamin E and those of the group B and could provide natural antioxidant compounds [3]. Amaranth (*Amaranthus* sp.) also belongs to the pseudocereals family. The nutritional quality of amaranth seed is higher than that of most cereal grains due to its protein quality, minerals, dietary fibers and lipid fraction which is rich in natural organic compounds that are involved in the metabolism of cholesterol and that could play an important role in lowering LDL-cholesterol in blood [4].

Wheat bread fortification could enable the development of a range of new baking products with enhanced nutritional value [5]. A large number of studies reported that bread supplemented with quinoa and amaranth flour had high nutritional value, while the consumers’ acceptance depends on the substitution level [4,5,6,7,8,9]. Generally, supplementation values up to 20% of quinoa or amaranth flour led to bread with enhanced nutritional, rheological and sensorial characteristics [4,5].

The combination of sourdough lactic acid bacteria and pseudocereals was successfully employed in the formulation of new products [10]. During sourdough fermentation, several beneficial transformations occur; by stimulating protein hydrolysis, the free aminoacid and bioactive peptide content are increased while the texture and aroma profile are improved [11]. Instead, the use of non-conventional yeast strains for bread dough fermentation has received relatively little attention. The increasing interest in artisanal products, as well as the demand for niche products with distinctive aroma profiles is leading to a renewed interest into the potential of non-conventional yeasts strains [12]. Non-conventional yeast strains can produce aroma profiles that are different from that produced by the commercial bakery strain [13]. Moreover, beside the impact on flavour, some non-conventional strains show exciting characteristics for bread fermentation, such as freeze tolerance, amylase activity or the ability to ferment complex sugars [14]. 

To the best of our knowledge, fermentation of quinoa and amaranth flours with aromatic yeast strains in order to obtain a preferment with high potential in enhancing nutritional, textural and sensorial features of white wheat bread was not investigated. Therefore, the purpose of our study was firstly, to optimize the fermentation of quinoa and amaranth flours with non-conventional yeast strains in order to obtain a preferment and secondly, to assess the preferment effect on bread quality.

## 2. Materials and Methods 

### 2.1. Materials 

Quinoa flour (QF, originated from Peru) and amaranth flour (AF, originated from Poland) were purchased from specialized stores in Romania. According to the producers, both flours were stone-mortar milled. Wheat flour (WF) was produced by a local mill (Goodmills Romania, Targu-Mures, Romania) and sold as type 650 according to ash content by Romanian classification (humidity 14.1%; wet gluten 29.3%; ash 0.63%; Falling Number 345s). Two biotypes of *Saccharomyces cerevisiae* yeast (Lallemand, Montréal, Canada) commercialized to their aromatic properties: a wine yeast strain (A17) and a beer yeast strain (A18), in active dry form, were used. 

All reagents were of analytical grade. Analytical reagents and chemicals were purchased from Sigma Aldrich (St. Louis, MO, USA), except for Boehringer-Mannheim enzymatic kits (starch, maltose/sucrose/glucose, ethanol) which were provided by R-Biopharm (Darmstadt, Germany).

### 2.2. Preferment Preparation

The aromatic yeast strains A17, A18 were rehydrated in warm water (35 °C) at a ratio water/yeasts = 15 for 15 min. From the rehydrated yeasts, two doses of 1% and 2% respectively, were taken and mixed with the corresponding amount of flours and water (Table 1) then incubated for 18 h at 30 °C (according to the instructions of use provided by the producer and supplier). The ratios between quinoa:wheat flours were 100:0 (A); 70:30 (B); 50:50 (C). Yeasts, amaranth and water amounts were relative to the mix of quinoa:wheat flours (Table 1).

### 2.3. Bread Making Process

All the preferments’ experimental variants (Table 1) were used in bread making. For 100 kg dough, the formula was: 26 kg preferment (P), 48 kg wheat flour (WF), 1 kg salt and 25 kg water. The dough was kneaded (Kemper mixer, WP Kemper GmbH, Rietberg, Germany) 2 min at I speed, 6 min at II speed at dough temperature of 24 °C, then it was pre-proofed for 90 min.

The dough (25–26 °C) was manually dived in pieces of 280 g, shaped in rectangular forms, proofed (30 °C, 30 min, 80% air relative humidity), baked in electrical oven (220 °C for 25 min), cooled and subjected to analysis. A Zanolli type of oven (Dr. Zanolli SRL, Verona) equipped with proofer was used. Bread samples were codified with the letter B by keeping the same numbers as in preferment.

### 2.4. Effect of Flours Substrate and Yeast Type on the Preferment Characteristics

#### 2.4.1. pH and Acidity

The pH value of each preferment was determined using a WTW pH-meter (Hanna Instruments, Vöhringen, Germany). Total titratable acidity (TTA) 10 g sample is diluted in 50 mL water, and then neutralized with 4 g/L (1 N) sodium hydroxide (NaOH) to pH 6.6. The resulting TTA value is expressed as milliliters of NaOH.

#### 2.4.2. Microbial Count and Viability

Preferment samples (1 mL) were suspended in 9 mL of sterile peptone saline diluent and homogenized in vortex; aliquots (0.1 mL) in serial 10-fold dilutions from each homogenate were spread on solid Yeast Extract–Peptone–Dextrose medium, YPD (Sigma-Aldrich) and incubated at 28 °C for 48 h. Colonies from all plates were analysed under an optical microscope (Zeiss 40X, Primo Star, Carl Zeiss Microscopy GmbH, Jena, Germany) in order to identify the specific microorganisms and counted using a colony counter (Colony Star 8500, Funke Gerber, Berlin, Germany). Microscopic structure of *Saccharomyces cerevisiae* strains was compared with microscopic images provided by Yeast Resource Center database, Informatics Platform Public Image Repository [15]. For the identification of dead cells, a suspension of cells is mixed with the same volume of a solution containing: methylene blue 200 mg, potassium dihydrogen phosphate 27.2 mg, disodium hydrogen phosphate 0.07 g/L of distilled water; in contrast to live cells, the dead cells stain red, allowing counting under a microscope with a Thoma camera (Paul Marienfeld GmbH & Co. KG, Lauda-Königshofen, Germany. The dead cells are calculated as percentage (%) from the total of counted cells.

#### 2.4.3. Sugars and Ethanol Determination

Sugars (starch, maltose/glucose) and ethanol concentrations from the preferment samples were determined enzymatically by using Starch UV colorimetric method (Roche, 748 343; Boehringer Mannheim), maltose/Sucrose/D-glucose UV colorimetric method (Roche, 748 327; Boehringer Mannheim), Ethanol UV colorimetric method (Roche, 668559; Boehringer Mannheim).

### 2.5. Bread Samples Textural and Nutritional Characteristics, Aromatic and Sensory Profile

#### 2.5.1. Texture Profile Analysis for Bread Samples

CT 3 Texture Analyzer (Brookfield Engineering Labs, Middleboro, MA, USA), equipped with 10 kg load cell and the TA11/1000 cylindrical probe (25.4 mm diameter AOAC Standard Clear Acrylic 21 g, 35 mm length) was used in a texture profile analysis test (40% target deformation, 1 mm s^−1^ test and post-test speed, 5 g trigger load, and 5 s recovery time). The bread samples were shaped as a conical trunk with 5 × 6.5 × 4 cm. The specific texture parameters were computed by Texture Pro CT V1.6 software (Brookfield Engineering Labs, Middleboro, MA, USA).

#### 2.5.2. Protein and Fiber Analyses

AACC Approved Methods [16] were used for crude fiber (32-07.01), while protein content were measured using the Kjeldahl method (46-11.02), nitrogen to protein conversion factor was 5.7. 

#### 2.5.3. Total Phenols and Antioxidant Activity 

The methods described by Chiș et al. [17] were used for total phenols and antioxidant capacity determination. Shortly, one gram of the bread sample was extracted three times with 100 mL acidified methanol (85:15 *v*/*v*, MeOH: HCl) by maceration under continuous stirring (Velp magnetic stirrer, Usmate (MB) – Italy) for 24 h. The filtrates were combined in a total extract, which was dried by using a vacuum rotary evaporator (Laborota 4010 digital rotary evaporator, Heidolph Instruments GmbH & Co. KG, Schwabach, Germany) at 40 °C. The dry residues were redissolved in 10 mL methanol (99.9% purity) and filtered through a 0.45 µm nylon filter (Millipore, Merck KGaA, Darmstadt, Germany). Total phenolics content from the extracts was determined by the Folin–Ciocalteu method. 100 µL of each extract was shaken for 1 min with 500 µL of Folin–Ciocalteu reagent and 6 mL of distilled water. After the mixture was shaken, 2 mL of 15% Na_2_CO_3_ was added and the mixture was shaken once again for 0.5 min. Finally, the solution was filled up with distilled water. Samples were kept in the dark for 2 h, and then, absorbance was read at 720 nm on a Shimadzu 1700 UV/visible spectrophotometer (Shimadzu Scientific Instruments, Kyoto, Japan). The total phenol content was read by plotting the gallic acid calibration curve (from 1 to 1500 µg/mL) and expressed as milligrams of gallic acid equivalents (GAE) per gram of dried extract. The equation or the gallic acid calibration curve was y = 1.02295x +0.08740, *R*^2^ = 0.99614.

The antioxidant activity was determined by using the radical DPPH (2,2-diphenyl-1- picrylhydrazyl) scavenging capacity assay as described by Chiș et al. [17]. The phenolic extracts (0.1 mL) were mixed with DPPH solution (3.9 mL), kept in the dark at ambient temperature, and the absorbance of the mixtures was recorded at 515 nm after exactly 30 min against methanol as blank. Negative control was prepared using 0.1 mL methanol and 3.90 mL of DPPH. The radical scavenging activity (RSA) was calculated according to the following Equation (1):

$$RSA[\%]=\frac{Abs_{DPPH}\cdot Abs_{Sample}}{Abs_{DPPH}}\cdot 100$$

where: *Abs _DPPH_* = absorbance of *DPPH* solution; *Abs _Sample_* = absorbance of the sample.

#### 2.5.4. Analysis of Macro and Microelements by Atomic Absorption Spectrophotometry

3 g bread was burned 10 h at 550 °C in furnace (Nabertherm B150, Lilienthal, Germany). The ash was dissolved in HCl 20% and was transferred by a final volume of 20 mL in a volumetric flask. The macroelements (K, Ca, Mg) and microelements (Fe, Cu, Zn and Mn) were determined by Atomic Absorption Spectrometer AAS (Varian 220 FAA equipment, Mulgrave, Victoria, Australia). Mix standard solutions (ICP Multielement Standard solution IV CertiPUR) were purchased from Merck (Merck KGaA, Darmstadt, Germany). All chemicals and solvents used in this study were of analytical grades. The results were expressed as related to the bread fresh weight.

#### 2.5.5. Extraction and Analysis of Volatile Compounds

The extraction of volatile compounds from 3 g of bread sample was performed using the in-tube extraction technique (ITEX). The analysis of volatile compounds was carried out on a gas-chromatograph mass spectrometer model GCMS QP-2010 (Shimadzu Scientific Instruments) model gas chromatograph–mass spectrometer equipped with a CombiPAL AOC-5000 auto-sampler. The volatile compounds were separated on a Zebron ZB-5 ms capillary column of 30 m × 0.25 mm i.d and 0.25 mm film thickness. The carrier gas was helium, 1 mL min^−1^ and the split ratio 1:5. The temperature program used for the column oven was: 30 °C (held for 5 min) rising to 110 °C with 4 °C min^−1^ and then heated to 250 °C with 15 °C min^−1^ and held for 5 min. The injector, ion-source and interface temperatures were set at 250 °C. The MS mode was electron impact (EI) at ionization energy of 70 eV. The mass range scanned was 40–400 m z^−1^. The volatile compounds were tentatively identified based on the spectra of reference compounds from National Institute of Standards (NIST) mass spectra libraries, namely NIST27 and NIST147 and verified by comparison with retention indices drawn from [18,19]. All peaks found in at least two of the three total ion chromatograms (TIC) were taken into account when calculating the total area of peaks (100%) and the relative areas of the volatile compounds.

### 2.6. Sensory Evaluation

The sensory characteristics of bread were evaluated by 50 trained sensory panels, asked to evaluate appearance, colour, taste, flavour, texture, and overall acceptability of the samples on a 5-point hedonic scale, ranging from 5 as like extremely to 1 as dislike extremely.

### 2.7. Statistical Analysis

The results of three independent (*n* = 3) assays performed with replicates each were expressed as means ± standard deviations. Data were analyzed by one way analysis of variance (ANOVA) using Minitab Statistical Software v.16 (Minitab Inc., State College, PA, USA), for each parameter, Tukey’s comparison tests were performed at a 95% confidence level. Principal component analysis was performed by the Unscrambler X v.10.5.1 software (Camo Software, Oslo, Norway).

## 3. Results and Discussion

### 3.1. Effect of Flours Substrate and Yeast Type on the Preferment Characteristics

**Figure 1:**
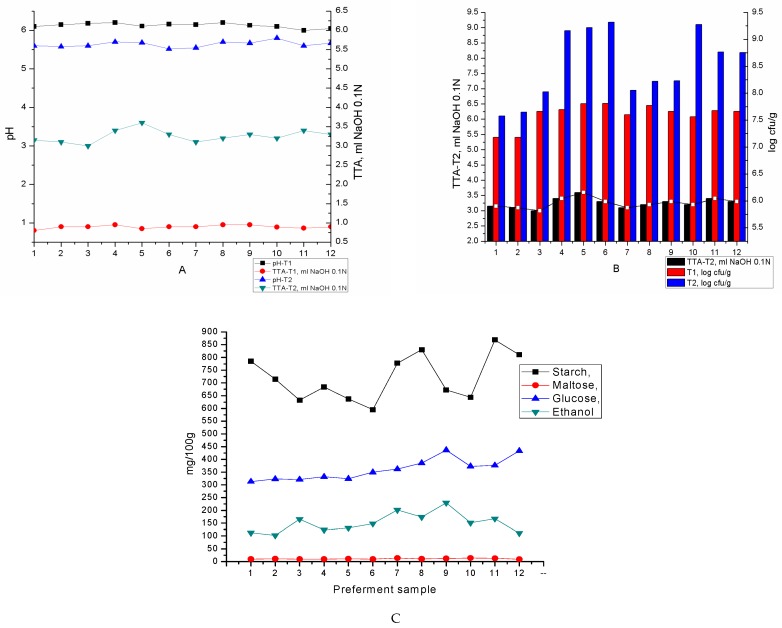
pH and total titratable acidity (TTA,%) variations (**A**), yeast counts (**B**) for preferment samples during fermentation period and carbohydrates conversion and ethanol formation after 18h of fermentation in pseudo-cereals preferment by aromatic yeast strains (**C**) (T1- sampling time before inoculation; T2- sampling time after 18h of fermentation. All determinations were performed in triplicate; For details, see Materials and methods; Each sample code is explained by sample name in Table 1).

Both yeast strains (A17, A18) were able to grow and slightly acidify quinoa slurry. The final cell densities of yeast strains in the fermented quinoa slurry had an average value of 2.2 × 10^7^ cfu/g in the case of 1% yeast cells inoculation (1 log cycle growth), while for the 2% yeast cells inoculation the cell growth during fermentation period was 2 log cycles (Figure 1B). Likewise, the yeast counts and the % of dead cells (an average of 1%) at the end of fermentation period, the same moment for the breadmaking beginning, show that both yeast strains could leaven the bread dough without conventional bakery yeast addition. The pH values of the preferments decreased from around 6.1 (before inoculation) to a minimum of 5.52 for sample P17.2.C, while the average value was 5.64 at the end of fermentation. With respect to TTA, the initial values started from 0.8 ml NaOH 0.1 N and increased to an average of 3.25 mL NaOH 0.1 N (Figure 1A). The yeast dosage (1 or 2%) and the ratio between flours (quinoa and wheat) didn’t significantly affect the acidification rate.

*Saccharomyces* yeast strains require media with sugars and nitrogen sources, for their growth and fermentative metabolism [12,13]. Slurries containing 50, 70, 100% quinoa flour and 3% amaranth flour could support aromatic yeast fermentative activity due to quinoa content in starch, fermentable sugars and nitrogen compounds. Also, quinoa was reported to have a slightly amylase activity [20,21] which together with the wheat flour - and yeast-associated enzymes that degrade carbohydrates lead to the corresponding release of maltose and glucose [13]. The main source of nitrogen in quinoa slurries are aminoacids, ammonium ions and di, tri-peptides [21]. It was reported that total aminoacid content in quinoa is higher than in barley, rice or wheat [2]. Next to nitrogen sources, minerals, and especially key metal ions, are important determinants of yeast performance. It has been shown that metal ions like Mg^2+,^ K^+^, Ca^2+,^ and Zn^2+^ have an impact on yeasts during brewing [13]. As reported by a large body of research, the main minerals in quinoa are potassium, phosphorus, and magnesium, while calcium and zinc are also present in relative high amounts [2]. Even if, quinoa was the main flour in preferment, amaranth flour also sustained the microbial growth and activity due to its rich composition in nitrogen compounds, minerals, vitamins and fermentable sugars [4]. Amaranth flour addition levels up to 3% didn’t affect the bread acceptability As results show in Figure 1C, glucose was the more abundant sugar in quinoa preferment after 18 h of fermentation, except for the non-depleted starch. The starch content in quinoa flours range between 58.1–64.2% [22] depending on the milling conditions. The intensity of starch hydrolysis may be influenced by the percentage of damaged starch present in quinoa flour after milling [23]. For both yeast strain biotypes used in this study, the average value of the residual starch after 18 h of fermentation in the selected quinoa slurries ranged between 7–8.5%. In this case, probably small saccharides are completely degraded within the first hour of fermentation and maltose remained to sustain fermentation. The aromatic yeast strains were capable to ferment maltose rapidly, after a very short period of adaptation, feature that is required for industrial processes [13,24].

Aromatic yeast strains, wine-type A17 and beer-type A18, revealed good performance in quinoa: wheat: amaranth slurries for carbohydrates conversion and ethanol production; only a slight difference was recorded in the case of beer-type A18 yeast strain, which was more effective in maltose/glucose formation and consequently in ethanol production. Generally, higher content of wheat flour in slurry led to higher ethanol content (P17.1.C, P17.2.C, P18.1.C), while the total absence of wheat flour (P17.1.A, P17.2.A, P18.1.A, P18.2.A) in preferment didn’t affect the aromatic yeast performance. Overall, as reported by previous research [12,24], both aromatic yeast strains indicated a good fermentative behaviour leading to a preferment with optimal features for breadmaking.

### 3.2. Bread Samples Textural and Nutritional Characteristics, Aromatic and Sensory Profile

Results of texture profile of bread samples obtained with quinoa preferment are shown in Table 2. Hardness is the principal mechanical characteristics for the consumer when eating solid foods and is defined as the force necessary to attain a given deformation [25]. The selected (100, 70, 50%) quinoa flour addition in preferment did not affect significantly (*p* > 0.05) the crumb hardness for the same yeast strain bread samples, even if yeast dosage varied from 1% to 2%. Bread samples with A18 yeast strain showed a significant reduction in hardness comparing to the samples obtained with A17 yeast (*p* < 0.05). In all cases, resilience, cohesiveness, springiness and gumminess recorded no significant variation (*p* > 0.05) for either quinoa flour addition or aromatic yeast type.

The gumminess and chewiness showed proportional trends with hardness. Cohesiveness reflects internal cohesion of the material and is largely of interest, with a preference for a high value so that during mastication the product does not disintegrate [20]. Generally, in wheat bread disulfide, hydrogen and ionic bonds maintain cohesiveness and contribute to gas retention during baking [26]. In the case of our study, the results for cohesiveness of bread samples, around of 0.54, indicated a more open and coarse crumb structure with larger cells and thicker cell walls due to the indirect baking method used and due to the high amount of water used in formulation. Crumb elasticity is described by springiness and resilience [25,27].

Resilience is defined as the ratio of the area under the curve of the second half of the first cycle to the first half, while the ideal bread springiness is 100%. For bread samples obtained in this study, these two parameters revealed good crumb elasticity; no statistically significant differences were recorded between samples (*p* > 0.05) for these two parameters. Springiness index ranged between 0.83 and 0.96, while resilience average value was 0.20 mJ. Crumb chewiness reflects the energy required to masticate food to a state ready for swallowing. Chewy foods tend to remain in the mouth without rapidly breaking up or dissolving [27].

All bread samples showed high content of protein, crude fiber and minerals (Table 3). A large body of research has reported the potential of pseudo-cereals, particularly of quinoa and amaranth flours, to contribute significantly to the enrichment of several bakery products in bioactive compounds [3,28]. The high protein content of quinoa flour (14%) and amaranth flour (17%) contributed significantly (*p* < 0.05) to the final protein content in bread samples, ranging from 11.50% to 13.72% as the proportion of quinoa flour varied. Also, the fiber content of bread samples revealed a similar trend (*p* < 0.05), with the highest content of 1.37% for sample with 100% quinoa flour in preferment.

The high content of quinoa and amaranth in minerals (Mg, P, K, Ca, Fe, Zn) is reflected, as we expected, in the mineral content of bread samples. The higher content of all minerals determined was recorded in bread samples obtained with 100% quinoa flour added in preferment (B17.1.A, B17.1.A; B18.1.A; B18.1.A). Significant differences (*p* < 0.05) were recorded between bread samples with different content of quinoa flour (Table 3). Similar results were reported by El-Sohaimy et al. [3] and Ibrahium [28] for bakery products enriched in quinoa. 

A large scientific literature states that quinoa and amaranth are very rich sources of minerals, more than three folds as compared to other cereals [2,22]. Moreover it is stated that yeasts could influence mineral availability by lowering the pH of the dough, thereby creating optimal conditions for wheat phytase activity [13] and so it is possible that aromatic yeast strains contributed also to the high mineral content.

Quinoa flour is used as a protein supplement in wheat flour and in the preparation of bakery products. The high and valuable content in protein, fiber, calcium, iron, zinc, copper and manganese are sufficient evidences to consider quinoa enriched bread as a complex food for a balanced diet. Due to its high content in fiber, quinoa could induce a low glycemic response, prolonging gastric distension, probably causing an increase in peptides associated with satiety [29]. Bread samples obtained with quinoa and amaranth flours registered good content of total polyphenols ranging from 314.48 to 636.73 mg GAE/100 g and high antioxidant activity, the variations between samples being statistically significant (*p* < 0.05) (Table 3). Other authors reported on bread enhanced with several ingredients with high antioxidant potential. For example, wheat bread supplemented with 8% turmeric reached 150.5 mg GAE/100 g total polyphenols and 79.8% antioxidant capacity [30] In the case of bread supplemented with 8% fenugreek flour a total polyphenols content of 379 mg GAE/100 g and 54% RSA were find [31]. If 2% green coffee extract was added in wheat bread total polyphenols were estimated to 75%, while RSA was 68.46% [32].

Repo-Carrasco et al. [33] reported that quinoa is a very good source of flavonoids findings superior content to those in flavonoid-rich berries such as lingonberry and cranberry. The same study concluded that the phenolic acid content of Andean indigenous crops was comparable to the content of these substances in oat, barley, corn and rice. It was also reported that quinoa presents higher antioxidant activity than amaranth [34]. Gorinstein et al. [35] concluded that along with buckwheat, quinoa has the highest contents of total polyphenols and the highest antioxidant potential among the cereals and pseudocereals investigated. The same research reported that quinoa proteins, also play a role in the overall antioxidant activity, while Flach Gewehr et al. [2] considered that the group of tocopherols (α,β,γ,δ) quantified in quinoa loaves contributed to the antioxidant activity.

GC-MS analysis of quinoa preferment obtained with aromatic yeast strains identified 30 volatile compounds (excluding long chain fatty acids) from different chemicals groups like as alcohols, aldehydes, esters, ketones, acids, terpenes, sulfur compound (Table S1, Supplementary data). For bread samples obtained with the above preferment, 22 volatile compounds were identified and 15 of them were sufficiently abundant (Table 4). Quinoa preferment revealed a larger number of volatile derivatives than the bread samples, with highest levels of aldehydes and ketones, compounds which were associated with earlier stages of fermentation [13,36]. 3-Methyl 1-butanol, 2-methyl-1-butanol, 3-methylbutanal and 2-methylbutanal, are usually associated with malt, alcohol, fermented odor and were found in high amounts in both aromatic yeast strains preferment. Principal Component Analysis (PCA) was used to analyse the volatile derivatives of bread samples (Figure S1A,B, Supplementary Data). Principal components (PC), namely PC 1 and PC 2 accounted for 54% and 21% of the total variance, respectively. From the third axis, the accumulated variance did not increase significantly. For this reason, this axis cannot be adopted. Therefore, maximum variance was obtained at 75 % (PC1 + PC2). In this plot, it can be observed that PC1 axis positively correlated with samples B17.2.A, B17.1.B, B17.1.C, and B18.1.C. On the other hand, PC2 axis was positively correlated only with samples B17.2.C, B17.2.B, B18.2.A. This confirm our results showing (Figure S1B) that 3-methyl-1-butanol, 2-methyl-1-butanol, 3-methylbutanal and 2-methylbutanal (marked as 1,2,4,5 on the plot) had the determinant contribution to the bread aroma profile.

Volatile esters are a particularly important class of aromatic compounds because these yeast-derived molecules are responsible for highly desired fruity aroma in fermentation products [13,37]. 

Our results showed the presence of numerous esters in quinoa preferment among which 3-methyl-1-butanol acetate (banana odor), ethyl decanoate (grape, fruit), ethyl octanoate (fruit) in highest amount for fermentation with A17 (wine yeast strain). In the case of A18 (beer yeast strain), the fermentative activity in quinoa preferment led to similar volatile compounds but with slightest content in fruity odours.

Compounds derived from lipid oxidation such as heptanal, octanal, nonanal, decanal are found in small amounts and only in preferment samples, suggesting a more effective activity in preferment than in bread samples due to the presence in highest quantities of lipids from pseudocereals.

It is now well known that the majority of the aroma compounds in bread are produced during baking due to the Maillard reactions but also recent studies [13,28] emphasized the role of the yeast strain and fermentation time on bread aroma profile. Both types of aromatic yeast strain for bakery purpose (wine and beer) gave a specific aroma profile to bread samples being responsible for the dominating compounds (alcohols, aldehydes, esters) which are derived from their metabolism. The aldehydes and their correspondents are formed inside the yeast cell from degradation of the flour amino acids via the Ehrlich pathway, while part of the alcohols, aldehydes and ketones could derive from oxidation of flour lipids and strongly influence the bread aroma profile [38]. Statistically significant differences (*p* < 0.05) were recorded between bread samples’ content in volatile derivatives as influenced by the yeast type and quinoa content (Table 4).

The most abundant were 3-methylbutanal and 2-methylbutanal (Table 4), representing between 60% and 78% of the total amount of volatile derivatives and without significant differences (*p* > 0.05) for the yeast dosage (1% or 2%) in bread samples. These two compounds are typically fermentation compounds likely formed via the Erhlich pathway in the yeast cell [38]. 3-methylbutanal and 3-methyl-1-butanol were considered as the most aroma active in wheat bread crumb [38]. Phenylethanol is also mentioned as one of the most important aroma compounds in bread crumb [39] and is considered that derives from catabolism of phenylalanine via the Erhlich pathway. The absence of aroma active lipid oxidation compounds, usually considered off-flavours, in bread samples might lead to higher consumer’s acceptance. This is correlated with the results of the sensory evaluation of the bread samples that are recorded values of overall acceptability between 4.2 and 4.6. Both, quinoa flour content and yeast strain did not influence significantly (*p* > 0.05) the scores as appear from the statistical analysis (Table 5).

## 4. Conclusions

Usually, aromatic yeasts are designed to ferment wheat substrates for baking purposes but identification of new substrates for these strains and consequently new formulations for dough could lead to diversified bakery products with improved nutritional qualities and specific sensorial properties. Quinoa and amaranth flour are indubitable important vectors for wheat bread nutritional enhancing. Aromatic yeast strains used in this study revealed good adaptability in pseudocereal flours used as substrate in order to obtain preferment for baking purpose. Significant differences (*p* < 0.05) in aroma compounds production were identified between the aromatic yeast strains used in the study.

## Figures and Tables

**Table 1:** Proportion of ingredients for the total amount of preferment used in 100 kg dough manufacturing.

Ingredients, kg	P 17.1.A	P 17.1.B	P 17.1.C	P 17.2.A	P 17.2.B	P 17.2.C	P 18.1.A	P 18.1.B	P 18.1.C	P 18.2.A	P 18.2.B	P 18.2.C
	*Sample Codes*											
	1	2	3	4	5	6	7	8	9	10	11	12
Quinoa flour (QF)	16.15	11.59	8.45	16.04	11.52	8.40	16.15	11.59	8.45	16.04	11.52	8.40
Wheat flour 650 (WF)	-	4.96	8.45	-	4.94	8.40	-	4.96	8.45	-	4.94	8.40
Amaranth Flour (AF)	0.49	0.49	0.49	0.49	0.49	0.49	0.49	0.49	0.49	0.49	0.49	0.49
Aromatic Yeast (AY 17 or AY 18)	0.16	0.16	0.16	0.32	0.32	0.32	0.16	0.16	0.16	0.32	0.32	0.32
Water	9.20	8.80	8.45	9.15	8.73	8.39	9.20	8.80	8.45	9.15	8.73	8.39
Total preferment (P), kg	26	26	26	26	26	26	26	26	26	26	26	26

The preferments samples codes represents: P-preferement; 17 or 18: yeast strain corresponding to the wine yeast strain (A17)and to the beer yeast strain (A18),respectively; 1 or 2: the yeast dose(%)inoculated in the slurry; A,B,C: the ratios (%) between quinoa:wheat flours 100:0:3(A), 70:30:3(B), 50:50:3(C); Sample’s codes (1 to 12) are used in the representation and explanation of the Figure 1.

**Table 2:** Texture profile analyses for bread samples.

Yeast Strain	Bread Samples	Hardness Cycle 1 [g]	Total work Cycle 1 [mJ]	Resilience [mJ]	Peak Stress [dyn/cm²]	Springiness Index [n.a.]	Cohesiveness [n.a.]	Gumminess [g]	Chewiness Index [g]
**A17**	**B 17.1.A**	2845 ± 549 ^a^	287.95 ± 105.9 ^a^	0.20 ± 0.04 ^a^	117432.3 ± 22649.20 ^a^	0.86 ± 0.04 ^a^	0.54 ± 0.07 ^a^	1506 ± 93 ^a^^,^^b^	128.6 ± 2.7 ^a^
	**B 17.1.B**	2348 ± 749 ^a^	242.8 ± 54.7 ^a^	0.24 ± 0.02 ^a^	96897.2 ± 30909.1 ^a^	0.89 ± 0.03 ^a^	0.59 ± 0.03 ^a^	1378 ± 387 ^a,bc^	123.2 ± 38.3 ^a^
	**B 17.1.C**	2835 ± 74 ^a^	287.8 ± 5.7 ^a^	0.18 ± 0.01 ^a^	117019.5 ± 3035.5 ^a^	0.83 ± 0.06 ^a^	0.50 ± 0.01 ^a^	1407 ± 14 ^a,b,c^	111.9 ± 16.0 ^a^
	**B 17.2.A**	2548 ± 489 ^a^	245.9 ± 28.4 ^a^	0.22 ± 0.04 ^a^	127275.8 ± 14879.6 ^a^	0.96 ± 0.04 ^a^	0.64 ± 0.06 ^a^	1634 ± 36 ^a^	156.6 ± 4.1 ^a^
	**B 17.2.B**	2347 ± 170 ^a^	280.2 ± 21.3 ^a^	0.16 ± 0.01 ^a^	117203.7 ± 8006.2 ^a^	0.83 ± 0.01 ^a^	0.53 ± 0.01^a^	1167 ± 118 ^a,bc^	163.8 ± 11.46 ^a^
	**B 17.2.C**	2488 ± 129 ^a^	295.8 ± 7.2 ^a^	0.16 ± 0.03 ^a^	124279.2 ± 15557.6 ^a^	0.86 ± 0.11 ^a^	0.52 ± 0.08 ^a^	1228 ± 35 ^a,bc^	183.0 ± 21 ^a^
**A18**	**B 18.1.A**	1991 ± 90 ^a^	242.6 ± 12.7 ^a^	0.26 ± 0.01 ^a^	99415.0 ± 4485.2 ^a^	0.92 ± 0.01 ^a^	0.58 ± 0.01 ^a^	1145 ± 57 ^a,b,c^	176.9 ± 11.1 ^a^
	**B 18.1.B**	1840 ± 166 ^a^	271.1 ± 22.1 ^a^	0.22 ± 0.00 ^a^	91873.4 ± 8299.4 ^a^	0.93 ± 0.01 ^a^	0.53 ± 0.01 ^a^	969 ± 61 ^b,c^	162.6 ± 14.1 ^a^
	**B 18.1.C**	1772 ± 233 ^a^	231.9 ± 17.3 ^a^	0.23 ± 0.04 ^a^	88502.1 ± 11654.4 ^a^	0.92 ± 0.0 ^a^	0.50 ± 0.04 ^a^	899 ± 193 ^c^	143.0 ± 30.8 ^a^
	**B 18.2.A**	1993 ± 11 ^a^	253.5 ± 5.1 ^a^	0.23 ± 0.09 ^a^	10984.5 ± 126.9 ^b^	0.90 ± 0.27 ^a^	0.56 ± 0.07 ^a^	1287 ± 43 ^a,b,c^	182.5 ± 9.5 ^a^
	**B 18.2.B**	1879 ± 27 ^a^	247.8 ± 6.9 ^a^	0.18 ± 0.05 ^a^	9876.2 ± 124.2 ^b^	0.86 ± 0.08 ^a^	0.55 ± 0.07 ^a^	1158 ± 72 ^a,b,c^	176.6 ± 10.5 ^a^
	**B 18.2.C**	1801 ± 25 ^a^	241.2 ± 14.1 ^a^	0.17 ± 0.07 ^a^	9012.3 ± 118.4 ^b^	0.85 ± 0.08 ^a^	0.52 ± 0.03 ^a^	1002 ± 6.36 ^b,c^	164.2 ± 16.5 ^a^

The bread samples codes represents: B-bread; 17 or 18: yeast strain corresponding to the wine yeast strain (A17)and to the beer yeast strain (A18),respectively; 1 or 2: the yeast dose(%)inoculated in the slurry; A,B,C: the ratios (%) between quinoa:wheat flours 100:0:3(A), 70:30:3(B), 50:50:3(C); All determinations were performed in triplicate; For details, see Materials and methods; a–c Mean values in the same column with different superscript letters differ significantly (*p* < 0.05).

**Table 3:** Nutritional characteristics for bread samples.

Yeast Strain	Bread Samples	Protein Content, g/100g dm	Crude Fiber, g/100g dm	Total Polyphenols (mg GAE/100g dm)	%RSA Antioxidant Activity	Ca, mg/100g dm	Mg, mg/100g dm	P, mg/100g dm	K, mg/100g dm	Fe, mg/100g dm	Cu, mg/100g dm	Zn, mg/100g dm	Mn, mg/100g dm
**A17**	**B 17.1.A**	13.72 ± 0.71 ^a^	1.31 ± 0.14 ^a^	592.12 ± 8.65 ^b^	63.17 ± 3.17 ^a,b^	93.87 ± 0.05 ^a^	120.58 ± 0.04 ^a^	198.45 ± 0.59 ^a^	151.31 ± 0.73 ^a^	5.78 ± 0.17 ^a^	0.98 ± 0.12 ^a^	0.80 ± 0.16 ^a,b^	1.15 ± 0.18 ^a,b^
	**B 17.1.B**	12.92 ± 0.38 ^a,b,c^	1.09 ± 0.15 ^a,b^	465.36 ± 7.45 ^d^	52.82 ± 3.54 ^a,b,c,d^	83.78 ± 0.10 ^c^	108.45 ± 1.13 ^d^	167.34 ± 0.42 ^d^	134.89 ± 1.06 ^c^	4.02 ± 0.25 ^b,c,d^	0.56 ± 0.07 ^b^	0.67 ± 0,09 ^a,b^	1.02 ± 0.22 ^a,b^
	**B 17.1.C**	11.81 ± 0.31 ^d,e,f^	0.72 ± 0.05 ^bc^	398.26 ± 2.60 ^f^	48.49 ± 2.65 ^c,d^	78.09 ± 0.23 ^de^	96.72 ± 1.23 ^f^	125.43 ± 0.2 ^g^	101.24 ± 0.50 ^f^	3.67 ± 0.09 ^d^	0.42 ± 0.12 ^b^	0.56 ± 0.15 ^b^	0.78 ± 0.21 ^a,b^
	**B 17.2.A**	12.85 ± 0.49 ^a,b,c,d^	1.21 ± 0.13 ^a^	507.76 ± 2.40 ^c^	65.73 ± 4.39 ^a^	95.67 ± 0.79 ^a^	118.03 ± 0.24 ^a,b^	188.23 ± 0.31 ^b^	143.04 ± 0.33 ^b^	5.09 ± 0.31 ^a,b,c^	1.05 ± 0.11 ^a^	0.96 ± 0.09 ^a^	1.15 ± 0.20 ^a,b^
	**B 17.2.B**	12.05 ± 0.39 ^c,d,e,f^	0.97 ± 0.22 ^a,b,c^	397.74 ± 10.29 ^f^	57.26 ± 3.99 ^a,b,c^	85.55 ± 1.15 ^c^	100.78 ± 0.89 ^e^	169.45 ± 0.55 ^c^	134.89 ± 0.61 ^c^	4.02 ± 0.17 ^b,c,d^	0.56 ± 0.19 ^b^	0.67 ± 0.14 ^a,b^	1.02 ± 0.24 ^a,b^
	**B 17.2.C**	11.50 ± 0.33 ^f^	0.68 ± 0.08 ^c^	314.48 ± 4.70 ^h^	42.07 ± 4.80 ^c^	75.09 ± 1.05 ^f^	89.89 ± 0.91 ^g^	115.06 ± 0.42^i^	109.14 ± 0.93 ^e^	3.99 ± 0.81 ^c,d^	0.51 ± 0.11 ^b^	0.61 ± 0.18 ^a,b^	0.69 ± 0.13 ^b^
**A18**	**B 18.1.A**	13.25 ± 0.26 ^ab^	1.37 ± 0.09 ^a^	591.35 ± 6.36 ^b^	66.45 ± 4.16 ^a^	90.57 ± 0.53 ^b^	115.88 ± 0.91 ^b^	200.45 ± 0.77 ^a^	140.81 ± 0.44 ^b^	5.19 ± 0.52 ^a,b^	0.98 ± 0.16 ^a^	0.67 ± 0.12 ^a,b^	1.23 ± 0.08 ^a^
	**B 18.1.B**	12.61 ± 0.22 ^b,c,d,e^	1.18 ± 0.06 ^a^	636.73 ± 7.78 ^a^	54.44 ± 6.11 ^a,b,c,d^	85.27 ± 0.99 ^c^	100.85 ± 0.95 ^e^	156.42 ± 0.88 ^e^	114.99 ± 0.42 ^d^	3.78 ± 0.37 ^d^	0.49 ± 0.09 ^b^	0.56 ± 0.10 ^b^	1.01 ± 0.18 ^a,b^
	**B 18.1.C**	11.72 ± 0.07 ^e,f^	0.99 ± 0.09 ^a,b,c^	338.27 ± 4.65 ^g^	51.38 ± 5.54 ^b,c,d^	76.89 ± 0.91 ^e,f^	90.52 ± 1.13 ^g^	117.38 ± 0.67 ^h^	98.54 ± 0.69 ^g^	3.67 ± 0.48 ^d^	0.37 ± 0.08 ^b^	0.67 ± 0.18 ^a,b^	0.89 ± 0.17 ^a,b^
	**B 18.2.A**	12.93 ± 0.10 ^a,b,c^	1.27 ± 0.12 ^a^	585.07 ± 4.32 ^b^	63.76 ± 5.44 ^a,b^	93.87 ± 0.51 ^a^	120.58 ± 0.64 ^a^	198.45 ± 1.22 ^a^	151.31 ± 0.91 ^a^	5.78 ± 0.27 ^a^	0.98 ± 0.19 ^a^	0.80 ± 0.08 ^a,b^	1.15 ± 0.22 ^a,b^
	**B 18.2.B**	12.03 ± 0.30 ^c,d,e,f^	1.04 ± 0.26 ^a,b,c^	445.32 ± 4.05 ^e^	59.89 ± 7.10 ^a,b,c^	88.28 ± 1.38 ^b^	111.15 ± 1.03 ^c^	157.47 ± 0.61 ^e^	141.89 ± 1.00 ^b^	4.66 ± 0.37 ^a,b,c,d^	0.69 ± 0.08 ^a,b^	0.47 ± 0.11 ^b^	1.12 ± 0.17 ^a,b^
	**B 18.2.C**	11.85 ± 0.19 ^d,e,f^	0.77 ± 0.08 ^b,c^	442.34 ± 4.69 ^e^	46.67 ± 4.97 ^c,d^	80.03 ± 1.16 ^d^	90.9 ± 0.90 ^g^	135.03 ± 0.98 ^f^	99.84 ± 1.05 ^f,g^	3.99 ± 0.50 ^cd,^	0.6 ± 0.12 ^b^	0.55 ± 0.10 ^b^	0.90 ± 0.13 ^b^

All determinations were performed in triplicate; For details, see Materials and Methods; a–i Mean values in the same column with different superscript letters differ significantly (*p* < 0.05)

**Table 4:** Volatile derivatives in bread samples.

No	Volatile Derivatives, %	B 17.1.A	B 17.1.B	B 17.1.C	B 17.2.A	B 17.2.B	B 17.2.C	B 18.1.A	B 18.1.B	B 18.1.C	B 18.2.A	B 18.2.B	B 18.2.C
	**Alcohols**												
**1**	3-Methyl-1-Butanol	17.93 ± 0.21 ^b,c,d^	16.23 ± 0.83 ^b^	12.73 ± 1.06 ^a^	21.48 ± 0.71 ^c^	20.21 ± 0.91 ^d,c^	21.59 ± 0.46 ^c^	14.8 ± 0.54 ^a,b^	12.03 ± 0.56 ^a^	16.26 ± 0.84 ^b^	19.89 ± 0.24 ^c,d,e^	17.23 ± 0.92 ^b,c,d^	16.79 ± 0.30 ^b,c^
**2**	2-Methyl 1-butanol	1.98 ± 0.19 ^a,b^	3.29 ± 0.69 ^b,c^	3.01 ± 0.60 ^b,c^	6.04 ± 0.80 ^d,e^	5.3 ± 0.38 ^d,e^	5.71 ± 0.37 ^d,e^	6.4 ± 0.61 ^e^	2.31 ± 0.54 ^a,b^	4.32 ± 0.66 ^c,d^	1.14 ± 0.22 ^a^	2.45 ± 0.52 ^a,b^	4.6 ± 0.51 ^c,d,e^
**3**	Phenylethanol	0.11 ± 0.05 ^a,b^	0.21 ± 0.11^b,c,d^	0.14 ± 0.05 ^a,b,c^	0.23 ± 0.08 ^c,d^	0.17 ± 0.06 ^b,c^	0.31 ± 0.05 ^d^	0.04 ± 0.04 ^a^	0.11 ± 0.09 ^a,b^	0.12 ± 0.05 ^a,b^	0.43 ± 0.10 ^e^	0.31 ± 0.07 ^d^	0.28 ± 0.06 ^d^
	**Aldehydes**												
**4**	3-Methylbutanal	51.3 ± 1.21 ^c,d,e^	54.86 ± 1.71 ^e^	58.98 ± 0.70 ^f^	54.03 ± 0.98 ^d,e^	51.74 ± 0.82 ^d,e^	50.43 ± 0.98 ^b,c,d^	47.02 ± 1.01 ^a,b^	54.41 ± 0.59 ^d,e^	59.71 ± 0.50 ^f^	54.02 ± 1.00 ^d,e^	47.55 ± 1.08 ^a,b,c^	46.32 ± 0.61 ^a^
**5**	2-Methylbutanal	23.56 ± 0.84 ^d^	19.54 ± 0.88 ^b,c^	20.47 ± 0.87 ^c,d^	22.9 ± 0.95 ^d^	16.65 ± 0.85 ^a,b^	18.05 ± 0.77 _a,b,c_	15.9 ± 0.37 ^a^	27.49 ± 0.74 ^c^	17.19 ± 0.89 ^a,b,c^	16.63 ± 0.47 ^ab^	14.67 ± 0.54 ^a^	15.09 ± 0.98 ^a^
**6**	Hexanal	0.29 ± 0.07 ^a^	0.59 ± 0.15 ^a,b^	0.45 ± 0.08 ^a,b^	2.45 ± 0.36 ^c^	0.65 ± 0.28 ^a,b^	0.3 ± 0.09 ^a^	0.93 ± 0.10 ^b^	0.37 ± 0.09 ^a,b^	0.5 ± 0.09 ^a,b^	0.87 ± 0.10 ^a,b^	0.56 ± 0.10 ^a,b^	0.67 ± 0.08 ^a,b^
**7**	Benzaldehyde	0.19 ± 0.05 ^a,b^	0.32 ± 0.10 ^b,d,e^	0.11 ± 0.05 ^a^	0.19 ± 0.04 ^a,b,c^	0.2 ± 0.06 ^a,b,c,d^	0.12 ± 0.06 ^a^	0.41 ± 0.12 ^e,f^	0.27 ± 0.05 ^b,c,d^	0.23 ± 0.07 ^a,b,c,d^	0.67 ± 0.09 ^g^	0.5 ± 0.09 ^f^	0.41 ± 0.07 ^e,f^
**8**	Phenylacetaldehyde	0.1 ± 0.05 ^a^	0.26 ± 0.11 ^b,c,d^	0.25 ± 0.08 ^b,c,d^	0.2 ± 0.05 ^a,b^	0.58 ± 0.10 ^f^	0.25 ± 0.07b ^c,d^	0.33 ± 0.10 ^c,d,e^	0.4 ± 0.09 ^e^	0.23 ± 0.06 ^b,c^	0.37 ± 0.06 ^d,e^	0.25 ± 0.07 ^b,c,d^	0.19 ± 0.04 ^a,b^
	**Others**												
**9**	2-Butanone	0.38 ± 0.07 ^a,b^	1.12 ± 0.16 ^d,e^	0.47 ± 0.13 ^a,b,c^	1.37 ± 0.09 ^e^	0.23 ± 0.07 ^a^	0.81 ± 0.14 ^b,c,d^	2.19 ± 0.32 ^f^	0.93 ± 0.14 ^c,d,e^	0.52 ± 0.09 ^a,b,c^	1.20 ± 0.38 ^d,e^	0.87 ± 0.05 ^b,c,d,e^	0.94 ± 0.16 ^c,d,e^
**10**	Acetophenone	0.14 ± 0.05 ^a,b,c^	0.54 ± 0.08 ^d,e^	0.04 ± 0.03 ^a^	0.62 ± 0.10 ^e^	0.15 ± 0.06 ^a,b,c^	0.08 ± 0.03 ^a,b^	0.41 ± 0.12 ^c,d,e^	0.18 ± 0.04 ^a,b,c^	1.19 ± 0.06 ^f^	0.38 ± 0.06 ^b,c,d,e^	0.4 ± 0.06 ^b,c,d,e^	0.29 ± 0.07 ^a,b,c,d^
**11**	Benzoic Acid	0.15 ± 0.09 ^a^	-	-	0.95 ± 0.11 ^b^	-	-	-	0.18 ± 0.06 ^a^	-	-	-	-
**12**	Styrene	0.41 ± 0.11 ^a,b,c^	0.82 ± 0.14 ^c,d^	0.29 ± 0.09 ^a^	0.76 ± 0.12 ^a,b,c^	0.7 ± 0.14 ^a,b,c,d^	0.94 ± 0.11 ^d^	0.54 ± 0.14 ^a,b,c,d^	0.33 ± 0.08 ^a^	0.32 ± 0.08 ^a^	0.49 ± 0.10 ^a,b,c^	0.36 ± 0.05 ^a,b^	0.3 ± 0.03 ^a^
**13**	Dimethyl trisulphide	0.16 ± 0.09 ^a,b^	0.8 ± 0.12 ^e^	0.4 ± 0.16 ^b,c,d^	0.27 ± 0.07 ^a,b,c^	0.65 ± 0.06 ^d,e^	0.47 ± 0.09 ^c, d^	0.49 ± 0.12 ^c,d,e^	0.09 ± 0.04 ^a^	0.43 ± 0.08 ^b,c,d^	0.67 ± 0.11 ^d,e^	0.45 ± 0.06 ^b,c,d^	0.37 ± 0.06 ^a,b,c,d^
**14**	β-Myrcene	0.87 ± 0.17 ^b^	0.25 ± 0.07 ^a^	0.25 ± 0.09 ^a^	0.4 ± 0.10 ^a,b^	0.24 ± 0.06 ^a^	0.33 ± 0.10 ^a,b^	0.34 ± 0.06 ^a,b^	0.19 ± 0.08 ^a^	0.16 ± 0.05 ^a^	0.33 ± 0.04 ^a,b^	0.28 ± 0.05 ^a^	0.16 ± 0.04 ^a^
**15**	Limonene	0.24 ± 0.10 ^a,b,c^	0.48 ± 0.14 ^d,e^	0.13 ± 0.07 ^a,b^	0.4 ± 0.14 ^c,d,e^	0.22 ± 0.07 ^a,b,c^	0.1 ± 0.05 ^a^	0.21 ± 0.04 ^a,b^	0.26 ± 0.09 ^a,b,c^	0.18 ± 0.09 ^a,b^	0.54 ± 0.08 ^e^	0.40 ± 0.07 ^c,d,e^	0.31 ± 0.08 ^b,c,d^

All determinations were performed in triplicate; For details, see Materials and Methods; a–f Mean values in the same row with different superscript letters differ significantly (*p* < 0.05)

**Table 5:** Scores of sensory evaluation of bread samples.

Bread Samples	Appearance	Texture	Color	Flavor	Taste	Overall Acceptability
**B17.1.A**	4.02 ± 0.52 ^a^	4.06 ± 0.15 ^a^	4.22 ± 0.98 ^a^	4.37 ± 0.36 ^a^	4.25 ± 0.14 ^a^	4.20 ± 0.87 ^a^
**B17.1.B**	4.04 ± 0.67 ^a^	4.12 ± 0.37 ^a^	4.37 ± 1.14 ^a^	4.37 ± 0.29 ^a^	4.54 ± 0.23 ^a^	4.28 ± 0.76 ^a^
**B17.1.C**	4.51 ± 0.79 ^a^	4.37 ± 0.49 ^a^	4.51 ± 1.20 ^a^	4.39 ± 0.41 ^a^	4.37 ± 0.30 ^a^	4.47 ± 0.78 ^a^
**B17.2.A**	4.43 ± 0.27 ^a^	4.41 ± 0.59 ^a^	4.47 ± 0.94 ^a^	4.35 ± 0.18 ^a^	4.12 ± 0.34 ^a^	4.39 ± 0.97 ^a^
**B17.2.B**	4.51 ± 0.63 ^a^	4.41 ± 0.68 ^a^	4.33 ± 0.88 ^a^	4.47 ± 0.29 ^a^	3.82 ± 0.47 ^a^	4.26 ± 0.92 ^a^
**B17.2.C**	4.55 ± 0.41 ^a^	4.55 ± 0.72 ^a^	4.51 ± 1.03 ^a^	4.67 ± 0.33 ^a^	4.20 ± 0.42 ^a^	4.49 ± 1.02 ^a^
**B18.1.A**	4.63 ± 0.78 ^a^	4.47 ± 0.84 ^a^	4.53 ± 0.87 ^a^	4.47 ± 0.46 ^a^	4.35 ± 0.27 ^a^	4.47 ± 0.85 ^a^
**B18.1.B**	4.61 ± 0.81 ^a^	4.67 ± 0.62 ^a^	4.63 ± 0.81 ^a^	4.49 ± 0.41 ^a^	4.29 ± 0.34 ^a^	4.53 ± 0.92 ^a^
**B18.1.C**	4.51 ± 0.75 ^a^	4.51 ± 0.71 ^a^	4.51 ± 0.76 ^a^	4.55 ± 0.53 ^a^	4.08 ± 0.30 ^a^	4.31 ± 1.12 ^a^
**B18.2.A**	4.61 ± 0.37 ^a^	4.51 ± 0.44 ^a^	4.43 ± 1.01 ^a^	4.49 ± 0.57 ^a^	4.22 ± 0.67 ^a^	4.49 ± 0.77 ^a^
**B18.2.B**	4.71 ± 0.46 ^a^	4.59 ± 0.68 ^a^	4.61 ± 1.10 ^a^	4.53 ± 0.60 ^a^	4.43 ± 0.75 ^a^	4.60 ± 0.73 ^a^
**B18.2.C**	4.43 ± 0.54 ^a^	4.43 ± 0.79 ^a^	4.47 ± 1.12 ^a^	4.24 ± 0.54 ^a^	3.97 ± 0.58 ^a^	4.29 ± 0.82 ^a^

All determinations were performed in triplicate; For details, see Materials and methods; a Same letter in the same column represent no significant differences between values (*p* > 0.05)

## Supplementary Materials

The following are available online at https://www.mdpi.com/2304-8158/8/10/443/s1, Figure S1: Principal component analysis (PCA) for volatile derivatives, Table S1: Volatile derivatives in preferment samples.

## References

[1] Boukid F., Folloni S., Sforza S., Vittadini E., Prandi B. (2018). Current trends in ancient grains-based foodstuffs: Insights into nutritional aspects and technological applications. Comprehensive Reviews. Food Sci. Food Saf..

[2] Flach Gewehr M., Pagno C.H., Danelli D., Marchi de Melo L., Hickmann Flôres S., Vogt de Jong E. (2016). Evaluation of the functionality of bread loaves prepared with quinoa flakes through biological tests. Braz. J. Pharm. Sci..

[3] El-Sohaimy S.A., Shehata M.G., Mehany T., Zeitoun M.A. (2019). Nutritional, physicochemical, and sensorial evaluation of flat bread supplemented with quinoa flour. Int. J. Food Sci..

[4] Sanz-Penella J.M., Wronkowska M., Soral-Smietana M., Haros M. (2013). Effect of whole amaranth flour on bread properties and nutritive value. LWT-Food Sci. Technol..

[5] Stikic R., Glamoclija D., Demin M., Biljana V.R., Zorica J., Dusanka M.O., Sven-Erik J., Mirjana M. (2012). Agronomical and nutritional evaluation of quinoa seeds (*Chenopodium quinoa* willd.) as an ingredient in bread formulations. J. Cereal Sci..

[6] Enriquez N., Peltzer M.A., Raimundi V., Tosi M.L. (2003). Characterization of the wheat and quinoa flour blends in relation to their bread making quality. J. Argent. Chem. Soc..

[7] Rodriguez-Sandoval E., Sandoval G., Cortes-Rodr’ıguez M. (2012). Effect of quinoa and potato flours on the thermomechanical and breadmaking properties of wheat flour. Braz. Arch. Bioltechnol..

[8] Milovanovic M., Demin M., Vucelic-Radovic B., Zarkovic B., Stikic R. (2014). Evaluation of the nutritional quality of wheat bread prepared with quinoa, buckwheat and pumpkin seedblends. J. Agric. Sci. Belgrade.

[9] Iglesias-Puig E., Monedero V., Haros M. (2015). Bread with whole quinoa flour and bifidobacterial phytases increases dietary mineral intake and bioavailability. LWT-Food Sci. Technol..

[10] Gobbetti M., Rizzello C.G., Di Cagno R., De Angelis M. (2014). How the sourdough may affect the functional features of leavened baked goods. Food Microbiol..

[11] Campo E., Del Arco L., Urtasun L., Oria R., Ferrer-Mairal A. (2016). Impact of sourdough on sensory properties and consumers’ preference of gluten-free breads enriched with teff flour. J. Cereal Sci..

[12] Aslankoohi E., Malaver B.H., Rezaei M.N., Steensels J., Courtin C.M., Verstrepen K.J. (2016). Non-conventional yeast strains increase the aroma complexity of bread. PLoS ONE.

[13] Struyf N., Van der Maelen E., Hemdane S., Verspreet J., Verstrepen K.J., Courtin C.M. (2017). Bread dough and baker’s yeast: An uplifting synergy. Comprehensive Review. Food Sci. Food Saf..

[14] Alves-Araújo C., Pacheco A., Almeida M.J., Spencer M.I., Leão C., Sousa M.J. (2007). Sugar utilization patterns and respiro-fermentative metabolism in the baker’s yeast Torulaspora delbrueckii. Microbiology.

[15] Yeast Resource Center Informatics Platform. Public Image Repository. http://images.yeastrc.org/imagerepo/searchImageRepoInit.do.

[16] AACC International (2000). Approved Methods of the American Association of Cereal Chemists.

[17] Chiș M.S., Păucean A., Stan L., Mureșan V., Vlaic R.A., Man S., Biriș-Dorhoi E.S., Muste S. (2018). *Lactobacillus plantarum* ATCC 8014 in quinoa sourdough adaptability and antioxidant potential. Rom. Biotechnol. Lett..

[18] The Pherobase—Database of Pheromones and Semiochemicals. http://www.pherobase.com/.

[19] Flavornet and Human Odor Space. http://www.flavornet.org/flavornet.html.

[20] Codină G.G., Franciuc S.G., Todosi-Sănduleac E. (2016). Studies on the influence of quinoa flour addition on bread quality. J. Food Eng..

[21] Deželak M., Zarnkow M., Becker T., Košir I.J. (2014). Processing of bottom-fermented gluten-free beer-like beverages based on buckwheat and quinoa malt with chemical and sensory characterization. J. Inst. Brew..

[22] Repo-Carrasco R., Espinoza C., Jacobsen S.E. (2003). Nutritional value and use of the andean crops quinoa (*Chenopodium quinoa*) and kañiwa (*Chenopodium pallidicaule*). Food Rev. Int..

[23] Park S.H., Maeda T., Morita N. (2005). Effect of whole quinoa flours and lipase on the chemical, rheological and bread making characteristics of wheat flour. J. Appl. Glycosci..

[24] Capozzi V., Makhoul S., Aprea E., Romano A., Cappellin L., Sanchez J.A., Spano G., Gasperi F., Scampicchio M., Biasioli F. (2016). PTR-MS Characterization of VOCs Associated with Commercial Aromatic Bakery Yeasts of Wine and Beer Origin. Molecules.

[25] Amigo J.M., Arantxa del Olmo A., Engelsen M.M., Lundkvist H., Engelsen S.B. (2016). Staling of white wheat bread crumb and effect of maltogenic α-amylases. Part 1: Spatial distribution and kinetic modeling of hardness and resilience. Food Chem..

[26] Valcárcel-Yamani B., Caetano da Silva Lannes S. (2013). Quality parameters of some Brazilian panettones. Braz. J. Pharm. Sci..

[27] Bourne M. (2002). Food Texture and Viscosity.

[28] Ibrahium M.I. (2015). Minerals bioavailability of wheat biscuit supplemented by quinoa flour. Middle East J. Agric..

[29] Mora A.C., Lares M., Gutiérrez R.H., Diaz R.O., Hernández M.S., Fernández-Trujillo J.P. (2013). Quinoa pasta influences some biochemical markers in consumers. Food.

[30] Lim H.S., Park S.H., Ghafoor K., Hwang S.Y., Park J. (2011). Quality and antioxidant properties of bread containing turmeric (*Curcuma longa* L.) cultivated in South Korea. Food Chem..

[31] Man S.M., Păucean A., Călian I.D., Mureșan V., Chiș M.S., Pop A., Mureșan A.E., Bota M., Muste S. (2019). Influence of Fenugreek Flour (*Trigonella foenum-graecum* L.) Addition on the Technofunctional Properties of Dark Wheat Flour. J. Food Qual..

[32] Mukkundur Vasudevaiah A., Chaturvedi A., Kulathooran R., Dasappa I. (2017). Effect of green coffee extract on rheological, physico-sensory and antioxidant properties of bread. Food Sci. Technol..

[33] Repo-Carrasco-Valencia R., Hellström J.K., Pihlava J.M., Mattila P.H. (2010). Flavonoids and other phenolic compounds in Andean indigenous grains: Quinoa (*Chenopodium quinoa*), kañiwa (*Chenopodium pallidicaule*) and kiwicha (*Amaran. Caudatus*). Food Chem..

[34] Vega-Galvez A., Miranda M., Vergara J., Uribe E., Puente L., Martınez E.A. (2010). Nutrition facts and functional potential of quinoa (*Chenopodium quinoa* willd.), an ancient Andean grain: A review. J. Sci. Food Agric..

[35] Gorinstein S., Medina Vargas O.J., Jaramillo N.O., Salas I.A., Martinez Ayala A.L., Arancibia-Avila P., Toledo F., Katrich E., Trakhtenberg S. (2007). The total polyphenols and the antioxidant potentials of some selected cereals and pseudocereals. Eur. Food Res. Technol..

[36] Annan N.T., Poll L., Sefa-Dedeh S., Plahar W.A., Jakobsen M. (2003). Volatile compounds produced by *Lactobacillus fermentum*, *Saccharomyces cerevisiae* and *Candida krusei* in single starter culture fermentations of Ghanaian maize dough. J. Appl. Microbiol..

[37] Callejo M.J., Navas J.J.G., Alba R., Escott C., Loira I., González M.C., Morata A. (2019). Wort fermentation and beer conditioning with selected non-Saccharomyces yeasts in craft beers. Eur. Food Res. Technol..

[38] Birch A.N., Petersen M.A., Arneborg N., Hansen Å.S. (2013). Influence of commercial baker’s yeasts on bread aroma profiles. Food Res. Int..

[39] Frasse P., Lambert S., Levesque C., Melcion D., Richard-Molard D., Chiron H. (1992). The influence of fermentation on volatile compounds in French bread crumb. Lebensm. Wiss. Technol..

