# Supplementary material for: Use of Pseudocereals Preferment Made with Aromatic Yeast Strains for Enhancing Wheat Bread Quality
Source: Foods. 2019 Sep 26;8(10):443. doi: 10.3390/foods8100443 (PMC6836221; doi:10.3390/foods8100443)
Supplement: Supplementary file 1 [file foods-08-00443-s001.pdf]

Table S1. Volatile derivatives in preferment samples.

Volatile Derivatives, %	P 17.1.A	P 17.1.B	P 17.1.C	P 17.2.A	P 17.2.B	P 17.2.C	P 18.1.A	P 18.1.B	P 18.1.C	P 18.2.A	P 18.2.B	P 18.2.C
Butanal, 3-methyl	2.99 ± 0.1	1.34 ± 0.02	1.94 ± 0.05	1.44 ± 0.03	3.13 ± 0.09	3.31 ± 0.8	0.92 ± 0.04	0.44 ± 0.02	0.58 ± 0.02	0.34 ± 0.01	0.33 ± 0.09	0.32 ± 0.03
Butanal, 2-methyl	1.06 ± 0.3	0.33 ± 0.02	0.51 ± 0.03	3.3 ± 0.9	0.72 ± 0.5	1.15 ± 0.4	0.37 ± 0.03	0.14 ± 0.01	0.28 ± 0.14	0.13 ± 0.02	0.11 ± 0.15	0.13 ± 0.25
2-Pentanone	0.05 ± 0.01	0.21 ± 0.01	0.15 ± 0.02	0.65 ± 0.1	0.73 ± 0.03	0.78 ± 0.02	0.09 ± 0.01	0.05 ± 0.02	0.07 ± 0.01	0.05 ± 0.01	0.19 ± 0.02	0.08 ± 0.01
Propanoic acid, ethyl ester	0.04 ± 0.01	0.07 ± 0.01	0.09 ± 0.02	0.03 ± 0.02	0.05 ± 0.01	0.03 ± 0.01	0.06 ± 0.01	0.03 ± 0.02	n.d	n.d	0.09 ± 0.1	0.05 ± 0.01
1-Butanol, 3-methyl	66.35 ± 2.4	72.16 ± 3.2	71.88 ± 3.7	13.63 ± 0.5	43.28 ± 1.2	42.28 ± 1.5	70.42 ± 3.6	75.32 ± 4.2	66.49 ± 2.8	65.65 ± 1.9	69.15 ± 1.6	77.35 ± 2.8
1-Butanol, 2-methyl	16.75 ± 1.3	18.25 ± 0.9	17.39 ± 1.02	15.78 ± 1.6	14.99 ± 2.7	17.8 ± 0.36	17.34 ± 2.01	17.84 ± 3.4	24.19 ± 2.6	23.77 ± 1.8	19.58 ± 0.9	14.68 ± 1.5
Propanoic acid, 2-methyl-, ethyl ester	0.09 ± 0.02	0.27 ± 0.15	0.11 ± 0.02	0.48 ± 0.13	0.57 ± 0.2	0.89 ± 0.3	0.15 ± 0.01	0.11 ± 0.1	0.23 ± 0.12	0.13 ± 0.03	0.23 ± 0.05	0.29 ± 0.02
Acetic acid, 2-methylpropyl ester	0.84 ± 0.01	0.36 ± 0.1	0.24 ± 0.15	1.09 ± 0.17	0.05 ± 0.02	0.77 ± 0.3	1.61 ± 1.4	0.39 ± 0.02	0.05 ± 0.02	0.67 ± 0.01	0.04 ± 0.01	0.49 ± 0.1
Butanoic acid, ethyl ester	0.44 ± 0.02	0.29 ± 0.01	0.54 ± 0.1	2.53 ± 0.9	1.55 ± 0.8	0.54 ± 0.02	0.6 ± 0.2	0.2 ± 0.02	0.43 ± 0.03	0.22 ± 0.04	0.07 ± 0.05	0.09 ± 0.01
1-Hexanol	0.13 ± 0.02	0.47 ± 0.01	0.28 ± 0.01	4.46 ± 1.2	3.84 ± 1.6	1.77 ± 0.9	0.06 ± 0.02	0.03 ± 0.01	0.38 ± 0.1	0.05 ± 0.02	0.27 ± 0.02	0.3 ± 0.01
1-Butanol, 3-methyl-, acetate	3.23 ± 0.4	0.22 ± 0.3	0.44 ± 0.02	0.26 ± 0.1	3.1 ± 0.5	0.39 ± 0.2	0.52 ± 0.1	0.28 ± 0.1	0.34 ± 0.2	0.15 ± 0.02	2.39 ± 0.9	0.07 ± 0.03
1-Butanol, 2-methyl-, acetate	0.44 ± 0.2	0.85 ± 0.03	0.67 ± 0.2	0.49 ± 0.15	0.24 ± 0.06	1.2 ± 0.85	9.88 ± 1.5	1.86 ± 0.5	0.02 ± 0.03	4.23 ± 1.6	0.43 ± 0.03	0.12 ± 0.02
Styrene	0.23 ± 0.01	0.17 ± 0.01	0.13 ± 0.02	0.14 ± 0.01	0.31 ± 0.15	0.08 ± 0.04	1.25 ± 0.3	0.37 ± 0.2	0.06 ± 0.01	0.55 ± 0.2	0.05 ± 0.1	0.27 ± 0.15
alpha.-Pinene	0.08 ± 0.02	0.36 ± 0.1	0.9 ± 0.25	1.31 ± 0.4	0.12 ± 0.03	0.04 ± 0.02	0.08 ± 0.1	0.08 ± 0.4	0.3 ± 0.26	0.1 ± 0.3	0.06 ± 0.01	0.24 ± 0.2
Benzaldehyde	0.15 ± 0.1	0.05 ± 0.02	0.09 ± 0.03	0.16 ± 0.1	1.16 ± 1.5	0.16 ± 0.04	0.19 ± 0.03	0.08 ± 0.03	0.23 ± 0.2	0.11 ± 0.1	0.75 ± 0.5	0.47 ± 0.04
Phenol	0.15 ± 0.08	n.d	0.04 ± 0.02	1.55 ± 0.1	0.18 ± 0.02	0.1 ± 0.1	n.d	0.51 ± 0.2	0.51 ± 0.2	0.79 ± 0.1	0.04 ± 0.02	n.d
Hexanoic acid, ethyl ester	0.75 ± 0.1	0.14 ± 0.1	0.71 ± 0.3	0.29 ± 0.15	0.36 ± 0.2	0.08 ± 0.1	0.14 ± 0.2	0.02 ± 0.02	0.06 ± 0.2	0.06 ± 0.1	0.03 ± 0.15	0.67 ± 0.2
3-Carene	0.12 ± 0.1	0.14 ± 0.15	0.06 ± 0.01	0.6 ± 0.2	0.2 ± 0.1	0.11 ± 0.02	0.08 ± 0.03	0.03 ± 0.01	0.73 ± 0.5	0.45 ± 0.1	0.39 ± 0.06	0.03 ± 0.01
p-Cymene	0.13 ± 0.02	0.04 ± 0.15	0.07 ± 0.01	0.62 ± 0.4	0.4 ± 0.14	0.71 ± 0.2	0.14 ± 0.02	0.25 ± 0.03	0.03 ± 0.01	n.d	0.12 ± 0.01	0.03 ± 0.02
D-Limonene	0.79 ± 0.2	1.01 ± 0.7	0.45 ± 0.1	2.9 ± 0.7	0.44 ± 0.1	0.28 ± 0.2	0.97 ± 0.1	0.13 ± 0.03	0.03 ± 0.01	0.18 ± 0.02	0.04 ± 0.01	0.26 ± 0.01

Acetophenone	0.22 ± 0.01	0.25 ± 0.1	0.1 ± 0.06	0.25 ± 0.1	0.18 ± 0.01	0.1 ± 0.1	0.03 ± 0.02	0.01 ± 0.01	0.05 ± 0.01	0.05 ± 0.01	0.4 ± 0.2	0.11 ± 0.1
Heptanoic acid, ethyl ester	0.05 ± 0.01	1.22 ± 0.2	0.04 ± 0.01	0.14 ± 0.01	0.25 ± 0.01	0.88 ± 0.2	0.29 ± 0.2	0.52 ± 0.3	0.36 ± 0.1	0.72 ± 0.2	0.16 ± 0.1	0.04 ± 0.2
Phenylethyl Alcohol	0.49 ± 0.2	0.02 ± 0.01	1.03 ± 0.3	1.88 ± 0.5	2 ± 0.9	0.23 ± 0.1	n.d	0.08 ± 0.05	0.14 ± 0.2	0.18 ± 0.1	0.02 ± 0.02	0.69 ± 0.3
Benzoic Acid	0.2 ± 0.15	0.33 ± 0.2	0.19 ± 0.1	0.31 ± 0.5	0.3 ± 0.1	0.07 ± 0.02	0.06 ± 0.01	0.08 ± 0.1	0.04 ± 0.1	0.02 ± 0.01	0.65 ± 0.01	0.13 ± 0.1
Benzoic acid, ethyl ester	0.05 ± 0.01	0.08 ± 0.01	1.07 ± 0.2	0.07 ± 0.02	0.1 ± 0.15	2.01 ± 0.6	0.69 ± 0.2	0.53 ± 0.35	0.77 ± 0.24	0.63 ± 0.2	0.01 ± 0.01	0.02 ± 0.01
Octanoic acid, ethyl ester	1.08 ± 0.1	n.d	0.03 ± 0.02	0.14 ± 0.06	0.14 ± 0.05	n.d	0.16 ± 0.2	0.03 ± 0.02	0.12 ± 0.2	0.03 ± 0.01	0.34 ± 0.1	0.53 ± 0.3
Carvone	0.04 ± 0.01	0.28 ± 0.1	0.37 ± 0.2	0.19 ± 0.1	0.24 ± 0.2	0.02 ± 0.01	0.07 ± 0.01	0.26 ± 0.1	0.04 ± 0.01	0.39 ± 0.1	0.21 ± 0.03	0.02 ± 0.01
Nonanoic acid, ethyl ester ethyl ester	0.02 ± 0.01	0.02 ± 0.01	0.26 ± 0.1	0.41 ± 0.2	0.29 ± 0.1	0.82 ± 0.1	0.08 ± 0.01	0.14 ± 0.1	0.02 ± 0.01	0.19 ± 0.1	0.33 ± 0.5	0.07 ± 0.01
Decanoic acid, ethyl ester	0.43 ± 0.2	0.34 ± 0.15	0.06 ± 0.02	0.31 ± 0.1	0.33 ± 0.2	0.3 ± 0.05	0.55 ± 0.08	0.04 ± 0.01	0.4 ± 0.2	0.07 ± 0.02	0.11 ± 0.09	0.09 ± 0.1
1-Dodecanol	0.23 ± 0.1	0.33 ± 0.1	0.94 ± 0.01	0.07 ± 0.1	0.22 ± 0.15	0.45 ± 0.2	0.46 ± 0.1	0.44 ± 0.2	0.08 ± 0.01	0.34 ± 0.1	0.26 ± 0.2	0.04 ± 0.01

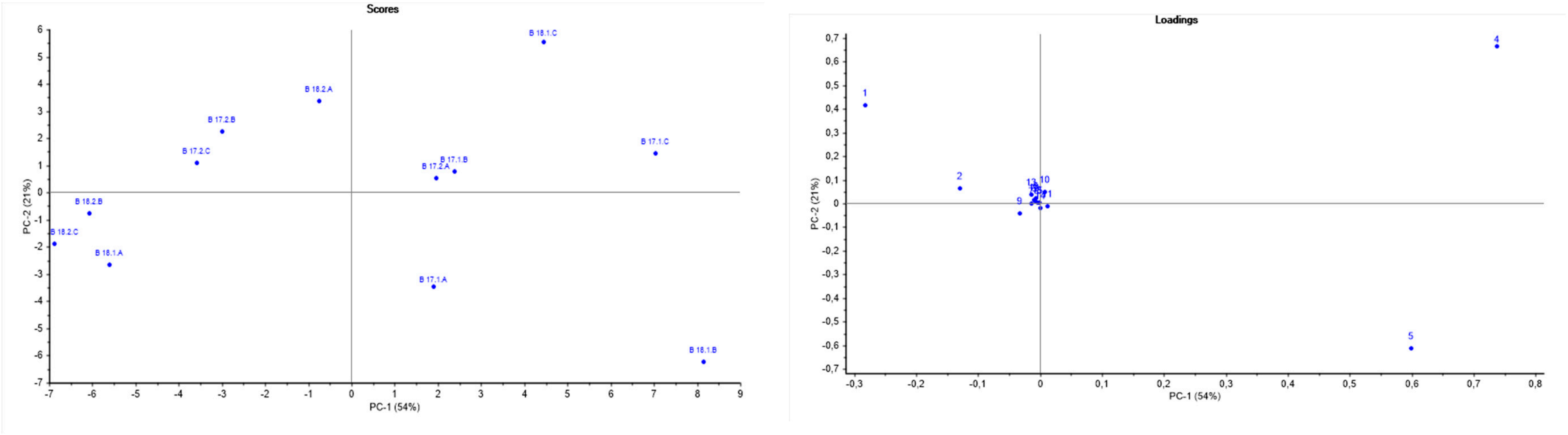

(**A**) (**B**)

Figure S1. Principal component analysis (PCA) for volatile compounds of bread samples – scores (A) and loadings (B) plots. Sample names are explained on Table 1 and volatile compounds indexes (1 to 15) are given on Table 4.
